# The endoscopic prediction model of simple endoscopic score for Crohn’s disease (SES-CD) as an effective predictor of intestinal obstruction in Crohn’s disease: A multicenter long-term follow-up study

**DOI:** 10.3389/fsurg.2022.984029

**Published:** 2022-09-09

**Authors:** Weimin Xu, Zhebin Hua, Yaosheng Wang, Yubei Gu, Jie Zhong, Long Cui, Peng Du

**Affiliations:** ^1^ Department of Colorectal Surgery, Xinhua Hospital, Shanghai Jiaotong University School of Medicine, Shanghai, China; ^2^ Department of Gastroenterology, Rui Jin Hospital, Affiliate to Shanghai Jiao Tong University, School of Medicine, Shanghai, China

**Keywords:** Crohn’s disease, Crohn’s disease activity index, simple endoscopic score for Crohn’s disease, intestinal obstruction, predictive value

## Abstract

**Background:**

The simple endoscopic score for Crohn's disease (SES-CD) is a widely used index to evaluate clinical and endoscopic activity. However, the association and predictive value of SES-CD for intestinal obstruction in Crohn's disease (CD) remains unclear. We aimed to establish the best cut-off indicators of SES-CD for early clinical intervention and subsequent prevention of intestinal obstruction in CD.

**Methods:**

Data on patients with CD evaluated at our institute from January 2016 to January 2022 were retrospectively collected. The SES-CD and Crohn's Disease Activity Index scores used in the analysis indicated the results of the first clinical and colonoscopy evaluations after hospitalization. The primary outcome was the occurrence of intestinal obstruction during admission and follow-up.

**Results:**

A total of 248 patients with a median follow-up time of 2 years [interquartile range: 1.0–4.0] were enrolled, of which 28.2% developed intestinal obstruction. An SES-CD score of 8 was the most significant threshold evaluation, and SES-CD ≥8 had the largest area under the receiver operating characteristic curve (0.705), with a sensitivity of 52.9% and specificity of 88.2% in predicting intestinal obstruction. Furthermore, SES-CD ≥8 had the greatest risk factor for intestinal obstruction (odds ratio: 7.731; 95% confidence interval: 3.901–15.322; *p* < 0.001) and significantly decreased the overall intestinal obstruction-free survival (*p* < 0.001).

**Conclusion:**

The SES-CD endoscopic prediction model could be an effective predictor of intestinal obstruction in patients with CD. More frequent follow-up and colonoscopic surveillance should be considered in patients with SES-CD score ≥8 to prevent the development of intestinal obstruction.

## Introduction

Crohn's disease (CD) is a chronic inflammatory bowel disease (IBD) characterized by recurrent relapse and remission, which markedly affects the clinical prognosis of patients. Chronic proliferative inflammation and fibrosis of the intestinal wall are the characteristic pathological changes of CD, which promote several serious complications, such as intestinal stenosis and obstruction, various fistulas, sinuses, abscesses, and perforations. Intestinal obstruction is one of the most common complications within the first 10 years of CD diagnosis (1, 2). Previous studies reported that approximately 30% of CD patients eventually developed end-stage intestinal fibrosis (3) and nearly 80% of patients underwent colectomy for fibrotic intestinal segments due to intestinal obstruction (4). However, there is currently no effective treatment for CD intestinal fibrosis (5). Therefore, it is particularly important for clinicians to develop effective methods that can predict intestinal obstruction in the course of the disease to provide early intervention.

The Crohn's Disease Activity Index (CDAI) has been widely considered the primary measure of evaluating the clinical disease activity in patients with CD for over 40 years (6). Colonoscopy is necessary for evaluating endoscopic activity in each patient. Although there is a significant correlation between Crohn's Disease Index of Severity and simple endoscopic score for Crohn's disease (SES-CD), SES-CD is a simple, reproducible, and easy-to-use endoscopic scoring system that has been widely used in clinical practice (7, 8). However, the association and predictive value of SES-CD for intestinal obstruction in CD remains unclear.

In this study, we aimed to determine the association between SES-CD and the development of intestinal obstruction and to establish the best cut-off indicators of SES-CD for early clinical intervention in preventing the occurrence of intestinal obstruction in CD.

## Methods

### Patients

The clinical data of patients with CD who received regular medical treatment or surgery from January 2016 to January 2022 at our institute were retrospectively identified and collected from a prospectively maintained, institutional review board-approved database (Chinese Database System for IBD) (9, 10). Patients diagnosed with CD who received regular follow-up and provided complete clinical data were included in this study. Patients who were lost to follow-up or had incomplete data were excluded. In this study, patients who underwent surgery were re-examined within 3 months, including fecal calprotectin, colonoscopy, and imaging examinations. Nonsurgical patients were followed up in outpatient clinics every month to observe changes in clinical symptoms, and endoscopy and imaging examinations were performed within 6 months. When clinical symptoms developed during follow-up, early and frequent surveillance was performed. Follow-up data for each patient were collected continuously at each center over a period. Among the follow-up data, complications of CD were considered for further analysis in the present study. The ethics committee of Xinhua Hospital reviewed and approved the study protocol (approval no. XHEC-D-2022-094).

### Evaluation of clinical and endoscopic activities

The CDAI and SES-CD scores of the first clinical and colonoscopic evaluations after hospitalization were used to assess the disease activity of CD (11). Detailed assessments of SES-CD and CDAI according to reports from previous research (7, 12) are presented in Supplementary Tables S1, 2. CDAI scores were evaluated by an independent IBD specialist according to the patients' physical signs (i.e., average daily temperature and extraintestinal manifestation), medication use (i.e., loperamide or opiate use for diarrhea), laboratory results (hematocrit), health conditions, and abdominal pain. CDAI-defined thresholds of 150, 221, and 450 were used for analysis, and CDAI ≥221 was considered as active CD based on the report of a previous study (12). The SES-CD scores were evaluated and recorded by two independent expert endoscopists who were blinded to the study. Higher endoscopic scores were used for further analysis when the endoscopic activity in the same affected area was inconsistent (13).

### Data evaluation

The primary outcome was the occurrence of intestinal obstruction during admission and follow-up. The diagnosis of CD was strictly based on endoscopic and histological examinations. CD-associated complications (intestinal obstruction, abscess, cellulitis, and fistula) were diagnosed based on the identified clinical manifestations, abdominal signs, and radiological examinations. The extent of CD was monitored for involvement in the small intestine, ileocolon, colon, and upper gastrointestinal tract (14). The use of medications (mesalamine, biologics, steroids, immunomodulators, and antibiotics) and laboratory results were also recorded.

### Statistical analysis

SPSS version 19.0 (IBM 2010, Chicago, IL, United States) and GraphPad Prism 8.0 (San Diego, CA, United States) were used for statistical analyses. Chi-square and Fisher's exact tests were used for univariate analyses. Multivariate logistic regression was used to determine the independent risk factors for intestinal obstruction. Receiver operating characteristic (ROC) curve analysis was performed to determine the predictive value of CDAI and SES-CD for intestinal obstruction. The Kaplan–Meier method with the log-rank test was used to compare the intestinal obstruction-free survival. All statistics were two-sided, with confidence intervals (CIs) set at 95%, and a *p*-value <0.05 was considered as significant.

## Results

### Baseline characteristics of patients

**Figure 1:**
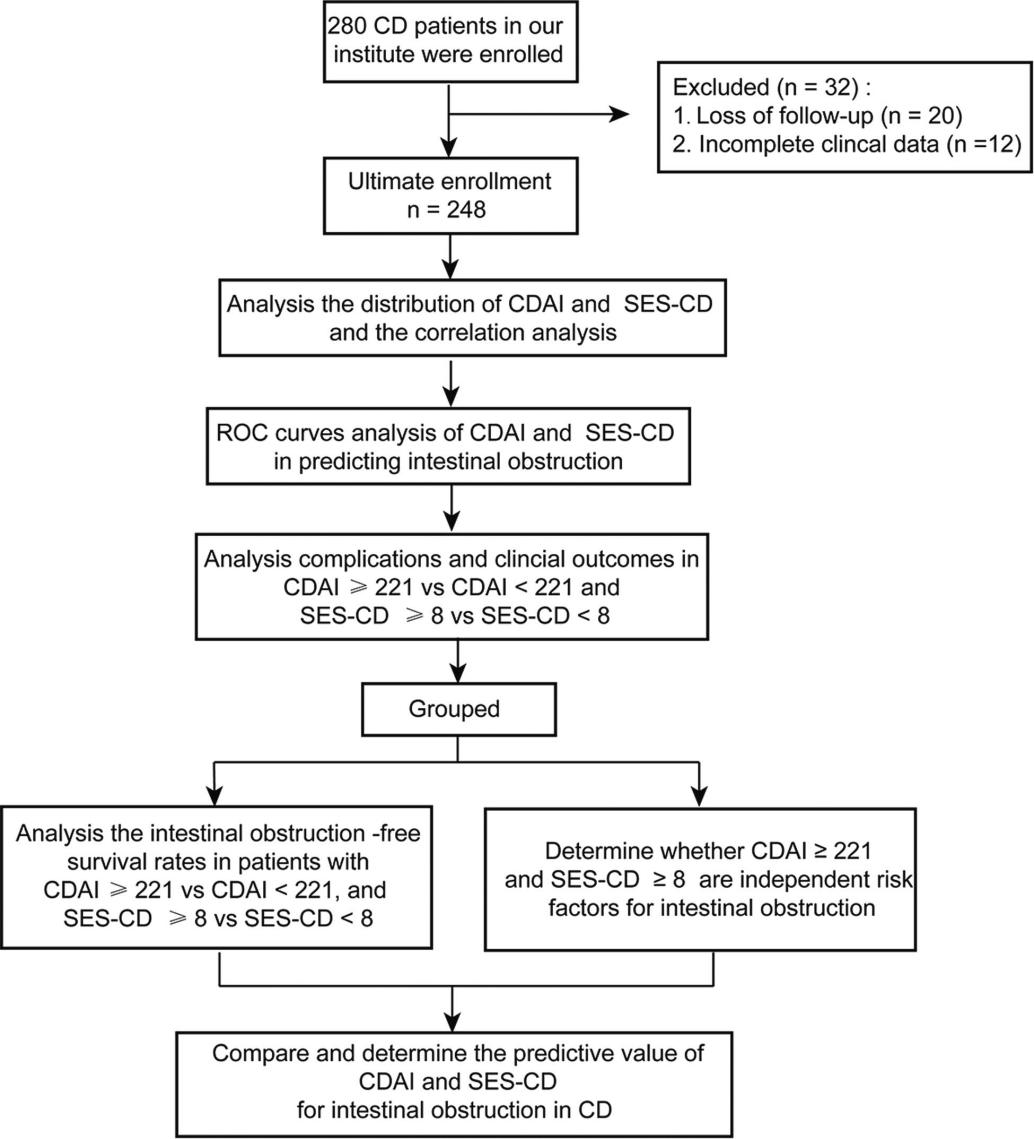
Schematic flow diagram of the present study.

As shown in Figure 1, 280 patients with CD were evaluated at our institute. Among them, 12 patients had incomplete clinical data and 20 were lost to follow-up. In total, 248 eligible CD patients with a median age of diagnosis of 33.0 years [interquartile range (IQR): 24.3–44.8] and a median follow-up time of 2 years (IQR: 1.0–4.0) were enrolled in this study. In the whole cohort, 114 (46.0%) patients had small intestinal lesions, 95 (38.3%) developed ileocolon CD, 35 (14.1%) had colon lesions, and 13 (5.2%) had upper gastrointestinal tract involvement (Table 1).

**Table 1:** Main baseline characteristics of patients.

Variables	All cases (*n* = 248)
Sex (male/female)	141/107
Age at diagnosis [year, median (IQR)]	33.0 (24.3–44.8)
Disease duration [year, median (IQR)]	3.0 (1.0–7.0)
Follow-up time [year, median (IQR)]	2.0 (1.0–4.0)
Extent of CD	
Small intestine, *n* (%)	114 (46.0)
Ileocolon, *n* (%)	95 (38.3)
Colon, *n* (%)	35 (14.1)
Upper gastrointestinal tract, *n* (%)	13 (5.2)
CDAI [score, mean ± SD]	280.1 ± 101.6
SES-CD [score, median (IQR)]	6.0 (3.0–7.0)
Medications	
Mesalamine, *n* (%)	129 (52.0)
Biologics, *n* (%)	80 (32.3)
Steroids, *n* (%)	54 (21.8)
Immunomodulators, *n* (%)	60 (24.2)
Antibiotics, *n* (%)	78 (31.5)
Laboratory data	
Hb (g/L, mean ± SD)	108.1 ± 21.3
Alb (g/L, mean ± SD)	31.8 ± 6.9
WBC (×10^9^/L, mean ± SD)	6.2 ± 3.0
CRP (mg/L, mean ± SD)	22.0 ± 28.5
ESR (mm/h, mean ± SD)	24.3 ± 20.0

CD, Crohn's disease; CDAI, Crohn's Disease Activity Index; SES-CD, simple endoscopic score for Crohn's disease; IQR, interquartile range; SD, standard deviation; ESR, erythrocyte sedimentation rate; CRP, C-reactive protein; Hb, hemoglobin; Alb, albumin; WBC, white blood cell.

**Figure 2:**
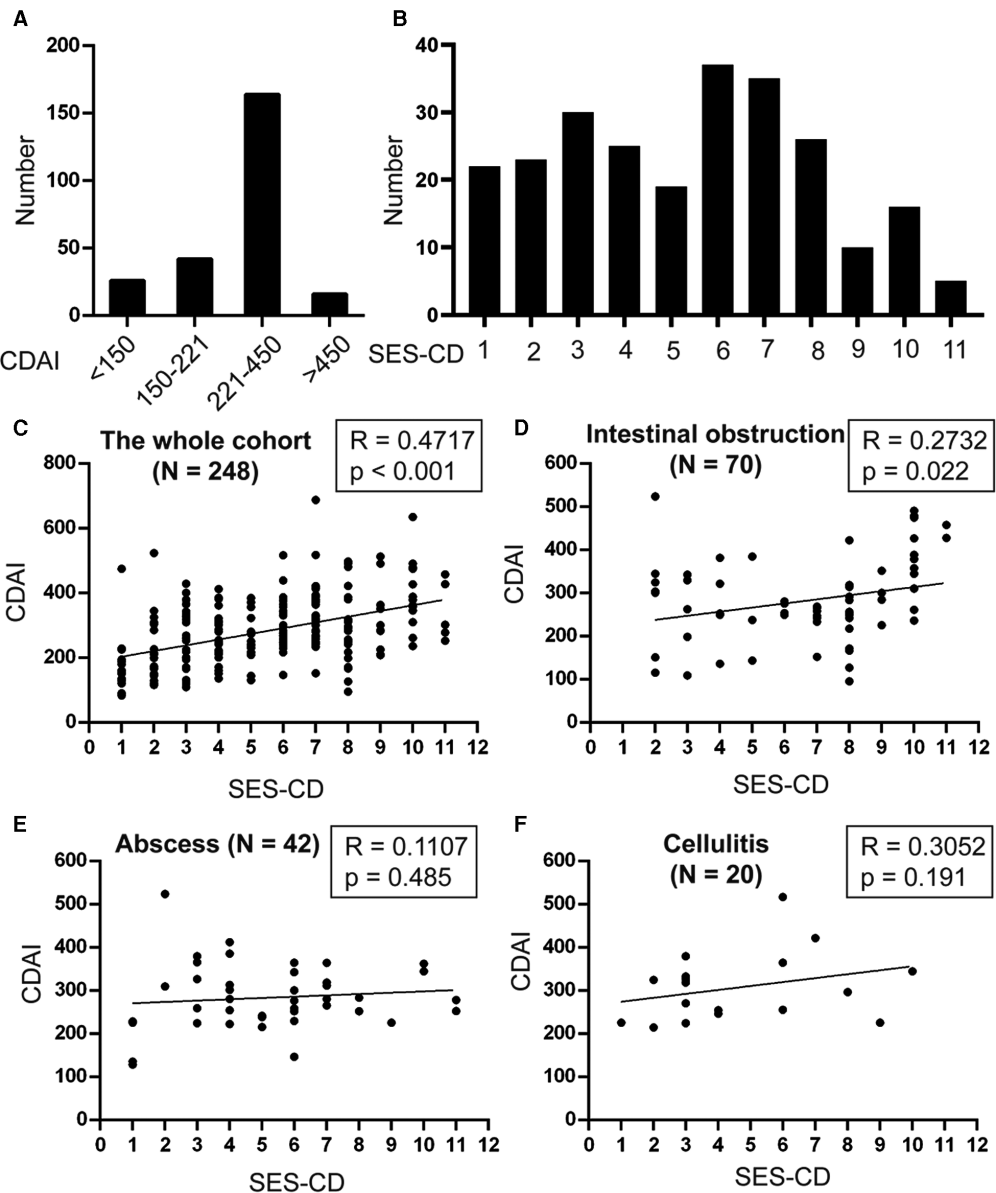
Analysis of the distribution and correlation of CDAI and SES-CD. The number of patients with different (**A**) CDAI and (**B**) SES-CD were analyzed. Spearman's correlation test was used to determine the correlation between CDAI and SES-CD in the (**C**) whole cohort, patients with (**D**) intestinal obstruction, (**E**) abscess, and (**F**) cellulitis.

We then analyzed the distribution of patients with different CDAI and SES-CD scores. We found that 180 (72.6%) patients were in the active CD stage (CDAI ≥ 221), and more than half of the patients (52.4%) had an SES-CD score of 6 or more (Figures 2A,B). We further demonstrated that the association between the CDAI and SES-CD scores was significant (R = 0.4717, *p* < 0.001, Figure 2C).

### Analysis of the complications and surgical intervention in CD

Intestinal obstruction was the most common complication in this study, which occurred in 70 (28.2%) CD patients. Furthermore, 42 (16.9%) patients developed abscesses, 20 (8.1%) had cellulitis, and 68 (27.4%) had fistulas (Table 2). A total of 92 (37.1%) patients received surgical intervention, of which 33 (35.9%) were due to medical treatment failure, 45 (48.9%) were due to acute or chronic intestinal obstruction, and 14 (15.2%) developed fistula perforation. In this study, the majority were laparoscopic surgeries, and more than 90% of patients achieved clinical remission after surgery (Table 2).

**Table 2:** Complications and clinical outcomes in CD patients.

Complications	Number (%)
Intestinal obstruction	70 (28.2)
Abscess	42 (16.9)
Cellulitis	20 (8.1)
Fistula	68 (27.4)
Surgical intervention	Number (%)
Surgery	92 (37.1)
Indications for surgery	
Medical treatment failure	33 (35.9)
Acute or chronic intestinal obstruction	45 (48.9)
Fistula perforation	14 (15.2)
Surgical approach	
Open	13 (14.1)
Laparoscopic	79 (85.9)
Postoperative outcomes	
Remission	84 (91.3)
Recurrence	8 (8.7)

CD, Crohn's disease.

### SES-CD threshold evaluation for predicting intestinal obstruction

**Figure 3:**
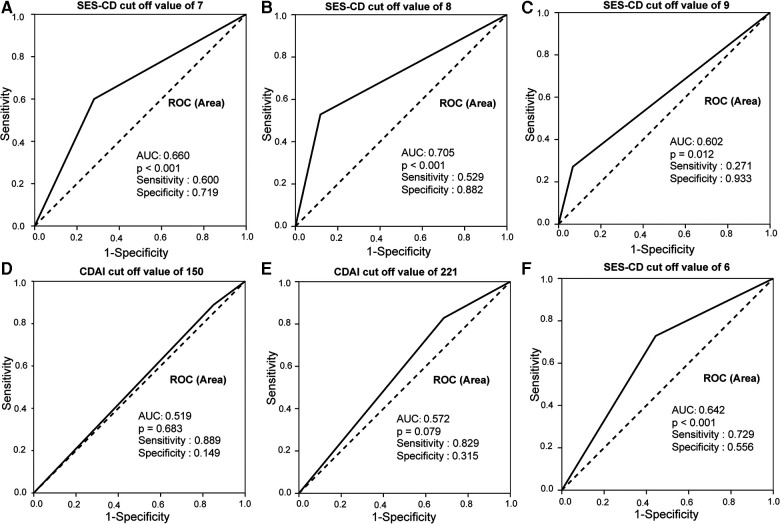
SES-CD and CDAI ROC curves in predicting intestinal obstruction in CD patients. (**A**–**C**) SES-CD ≥8 had the most significant AUC of 0.705 with a sensitivity of 52.9% and specificity of 88.2%. (**D–F**) CDAI ≥221 had the biggest area under AUC of 0.572 with a sensitivity of 82.9% and specificity of 31.5.

To determine the best threshold value of SES-CD for the indication of intestinal obstruction, an ROC curve was generated. As shown in Figures 3A–F, SES-CD ≥8 had the most significant area under the ROC curve (AUC) (0.705), with a sensitivity of 52.9% and a specificity of 88.2% (*p* < 0.001), while CDAI showed no significance in predicting the occurrence of intestinal obstruction (*p* = 0.079). This result indicated that an SES-CD score of 8 can predict the occurrence of intestinal obstruction more effectively than clinical activity.

### Analysis of the complications in patients with different endoscopic and clinical activity scores

Based on the results, a CDAI score of 221 and an SES-CD score of 8 were used to further analyze the complications and outcomes of the patients. First, we indicated that patients with SES-CD ≥8 were more likely to develop intestinal obstruction (*p* < 0.001) and undergo surgical intervention (*p* < 0.001), although patients with active CD were also prone to develop intestinal obstruction (*p* = 0.023), abscess (*p* = 0.004), and cellulitis (*p* = 0.019) (Table 3). Furthermore, we found that there was a significant correlation between CDAI and SES-CD scores for intestinal obstruction (R = 0.2732, *p* = 0.022); however, no significant correlation was observed between these two for abscess and cellulitis (Figures 2D–F).

**Table 3:** Analysis of the clinical outcomes in patients with different CDAI and SES-CD scores.

Variables	Clinical disease activity		*p*-value	Endoscopic disease activity		*p*-value
	CDAI < 221	CDAI ≥ 221		SES-CD < 8	SES-CD ≥ 8	
Intestinal obstruction						
No	56 (82.4)	122 (67.8)	0.023^a^	157 (82.6)	21 (36.2)	<0.001^a^
Yes	12 (17.6)	58 (32.2)		33 (17.4)	37 (63.8)	
Abscess						
No	64 (94.1)	142 (78.9)	0.004^a^	155 (81.6)	51 (87.9)	0.259^a^
Yes	4 (5.9)	38 (21.1)		35 (18.4)	7 (12.1)	
Cellulitis						
No	67 (98.5)	161 (89.4)	0.019^b^	173 (91.1)	55 (94.8)	0.581^b^
Yes	1 (1.5)	19 (10.6)		17 (8.9)	3 (5.2)	
Fistula						
No	49 (72.1)	131 (72.8)	0.910^a^	138 (72.6)	42 (72.4)	0.974^a^
Yes	19 (27.9)	49 (27.2)		52 (27.4)	16 (27.6)	
Surgery						
No	40 (58.5)	116 (64.4)	0.414^a^	135 (71.1)	21 (36.2)	<0.001^a^
Yes	28 (41.2)	64 (35.6)		55 (28.9)	37 (63.8)	

^a^ Chi-square test.

^b^ Fisher's exact test.

CDAI, Crohn's Disease Activity Index; SES-CD, simple endoscopic score for Crohn's disease.

### SES-CD ≥8 as the most significant independent risk factor for intestinal obstruction

To further determine whether SES-CD ≥8 and clinical disease activity could contribute to the development of intestinal obstruction in CD, univariate analysis was performed. Disease duration (*p* = 0.028), CDAI score (*p* = 0.023), SES-CD (*p* < 0.001), use of immunomodulators (*p* = 0.046), and albumin level (Alb, *p* = 0.019) were significantly associated with intestinal obstruction (Table 4). Multivariate logistic regression further demonstrated that CDAI ≥221 [odds ratio (OR): 2.818; 95% CI: 1.218–6.519; *p* = 0.016], SES-CD ≥8 (OR: 7.731; 95% CI: 3.901–15.322, *p* < 0.001), and albumin < 35 g/L (OR: 2.501; 95% CI: 1.233–5.073, *p* = 0.011) were independent risk factors for intestinal obstruction. This result indicated that patients with SES-CD ≥8 were at the highest risk for the development of intestinal obstruction (Table 5).

**Table 4:** Univariate analysis of risk factors for intestinal obstruction in CD patients.

Variables	Nonintestinal obstruction group (*N* = 178)	Intestinal obstruction group (*N* = 70)	*p*-value
Sex, *n* (%)			
Male	100 (56.2)	41 (58.6)	0.732^a^
Female	78 (43.8)	29 (41.4)	
Age at diagnosis, *n* (%)			
≤35 years	100 (56.2)	34 (48.6)	0.279^a^
>35 years	78 (43.8)	36 (51.4)	
Disease duration, *n* (%)			
<5 years	116 (65.2)	35 (50.0)	0.028^a^
≥5 years	62 (34.8)	35 (50.0)	
CDAI scores, *n* (%)			
CDAI < 221	56 (31.5)	12 (17.1)	0.023^a^
CDAI ≥ 221	122 (68.5)	58 (82.9)	
SES-CD scores, *n* (%)			
SES-CD < 8	157 (88.2)	33 (47.1)	<0.001^a^
SES-CD ≥ 8	21 (11.8)	37 (52.9)	
Extent of CD Small intestine, *n* (%)			
No	100 (56.2)	34 (48.6)	0.279^a^
Yes	78 (43.8)	36 (51.4)	
Ileocolon, *n* (%)			
No	107 (60.1)	46 (65.7)	0.414^a^
Yes	71 (39.9)	24 (34.3)	
Colon, *n* (%)			
No	151 (84.8)	62 (88.6)	0.446^a^
Yes	27 (15.2)	8 (11.4)	
Medications Mesalamine, *n* (%)			
No	90 (50.6)	29 (41.4)	0.195^a^
Yes	88 (49.4)	41 (58.6)	
Immunomodulators, *n* (%)			
No	141 (79.2)	47 (67.1)	0.046^a^
Yes	37 (20.8)	23 (32.9)	
Steroids, *n* (%)			
No	148 (83.1)	51 (72.9)	0.067^a^
Yes	30 (16.9)	19 (27.1)	
Biologics *n* (%)			
No	116 (65.2)	52 (74.3)	0.167^a^
Yes	62 (34.8)	18 (25.7)	
Antibiotics, *n* (%)			
No	128 (71.9)	42 (60.0)	0.069^a^
Yes	50 (28.1)	28 (40.0)	
Laboratory data Albumin, *n* (%)			
≥35 g/L	58 (32.6)	34 (48.6)	0.019^a^
<35 g/L	120 (67.4)	36 (51.4)	
Hb, *n* (%)			
≥110 g/L	84 (47.2)	42 (60.0)	0.069^a^
<110 g/L	94 (52.8)	28 (40.0)	
WBC, *n* (%)			
<10 × 10^9^/L	163 (91.6)	65 (92.9)	0.738^a^
≥10 × 10^9^/L	15 (8.4)	5 (7.1)	
CRP, *n* (%)			
<10 mg/L	85 (47.8)	38 (34.3)	0.354^a^
≥10 mg/L	93 (52.2)	32 (45.7)	
ESR, *n* (%)			
<20 mm/h	91 (51.1)	41 (58.6)	0.290^a^
≥20 mm/h	87 (48.9)	29 (41.4)	

^a^ Chi-square test.

CD, Crohn's disease; CDAI, Crohn's Disease Activity Index; SES-CD, simple endoscopic score for Crohn's disease; ESR, erythrocyte sedimentation rate; CRP, C-reactive protein; Hb, hemoglobin; WBC, white blood cell.

**Table 5:** Multivariate logistic regression analysis of risk factors for intestinal obstruction in CD patients.

Variable	Univariate			Multivariate		
	Odds ratio	95% CI	*p*-value	Odds ratio	95% CI	*p*-value
Disease duration ≥5 years	1.871	1.068–3.278	0.029	1.525	0.785–2.963	0.213
CDAI ≥ 221	2.219	1.105–4.456	0.025	2.818	1.218–6.519	0.016
SES-CD ≥ 8	8.382	4.359–16.120	<0.001	7.731	3.901–15.322	<0.001
Without immunomodulators	1.865	1.007–3.454	0.047	1.522	0.725–3.195	0.267
Alb < 35 g/L	1.954	1.112–3.434	0.020	2.501	1.233–5.073	0.011

CD, Crohn's disease; CDAI, Crohn's Disease Activity Index; SES-CD, simple endoscopic score for Crohn's disease; CI, confidence interval; Alb, albumin.

**Figure 4:**
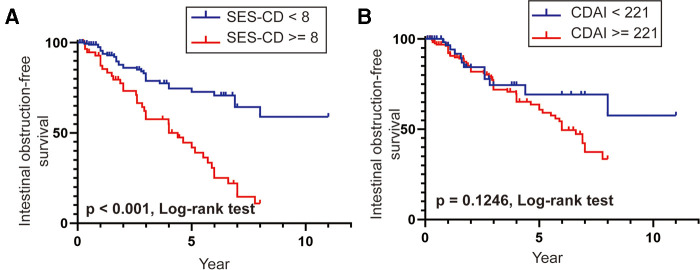
(**A,B**) Intestinal obstruction-free survival rates in patients with SES-CD ≥8 vs. SES-CD <8 and CDAI ≥221 vs. CDAI <221.

We further analyzed the overall intestinal obstruction-free survival in patients with different endoscopic and clinical activity scores. The results of the Kaplan–Meier method with the log-rank test indicated that the overall intestinal obstruction-free survival was significantly lower in patients with SES-CD ≥8 than that in patients with SES-CD <8 (*p* < 0.001, Figure 4A), which demonstrated that patients with SES-CD ≥8 were more likely to develop intestinal obstruction earlier in the disease duration. However, there was no statistically significant difference in the intestinal obstruction-free survival in patients in remission or with a clinical activity status (Figure 4B). Taken together, these findings demonstrate that the endoscopic prediction model of SES-CD could be an effective predictor of intestinal obstruction in patients with CD.

## Discussion

The SES-CD indices are widely used by clinicians to evaluate the endoscopic disease activity of CD. Although stenosis is one of the items of SES-CD evaluation, whether SES-CD is an effective indicator of intestinal obstruction has not been clarified yet. Here, we demonstrated a significant correlation between intestinal obstruction and SES-CD. We then identified the most significant threshold evaluation for SES-CD and demonstrated that the SES-CD score of 8 had the best predictive value for intestinal obstruction. We further indicated that SES-CD ≥8 was the most risk contributing factor for intestinal obstruction and significantly decreased intestinal obstruction-free survival time. These data indicate that the endoscopic prediction model of SES-CD could be an effective predictor of intestinal obstruction in CD, outperforming the clinical disease activity. Therefore, more frequent follow-up and colonoscopic surveillance should be considered in patients with SES-CD ≥8, especially in those with mild clinical manifestations but high endoscopic activity.

Intestinal obstruction is one of the most common complications of CD. Activation of fibroblasts leads to abnormal deposition of the extracellular matrix in the mucosa and submucosa, which is an important cause of fibrosis and stenosis formation, leading to intestinal obstruction (15). In intestinal inflammation, various signaling pathways including TGF-b/SMAD, Wnt/β-catenin, and Rho-ROCK can promote the conversion of fibroblasts into myofibroblasts. Myofibroblasts have fibrotic phenotypes such as proliferation, matrix deposition, and anti-apoptosis, which can eventually drive intestinal fibrosis (16). However, currently, no effective antifibrotic therapies exist for intestinal fibrosis to prevent the development of intestinal obstruction. Thus, it is important to explore effective clinical indicators to perform earlier and more frequent surveillance of CD patients.

Previous studies have reported that SES-CD can indicate the endoscopic remission of vedolizumab and ustekinumab in CD patients (17–19). SES-CD has also been used to evaluate the efficacy and safety of the simultaneous treatment with two biological medications for refractory CD (20). Recently, a study reported that SES-CD >2 on the first postoperative ileocolonoscopy was a significant risk factor for clinical recurrence after surgery. Additionally, SES-CD of 1 and 5 were the appropriate cut-off values in predicting the clinical recurrence of small intestine-dominant and colon-dominant CD, respectively (21). However, data on the relationship between SES-CD and intestinal obstruction are limited. In this study, we first reported that SES-CD ≥8 had the best predictive value and was the most significant risk factor for intestinal obstruction.

The CDAI is commonly used in clinical practice to assess the disease activity in patients with CD. Although the validity, reliability, and responsiveness of the CDAI are well defined (6, 22, 23), it has several limitations as an outcome measurement system in clinical practice. The evaluation of some items in the CDAI depends on patients' subjectivity, and some items lack a clear definition in standard (12). Moreover, CDAI reflects systemic inflammation and lacks specificity, whereas SES-CD focuses on intestinal inflammation. Thus, patients with higher SES-CD scores are prone to more severe intestinal inflammation, leading to further activation of intestinal fibroblasts, which could, to some extent, explain why SES-CD outperforms CDAI in predicting intestinal obstruction. As we previously reported, Yes-associated protein (YAP) and transcriptional coactivator with PDZ-binding motif (TAZ), the transcriptional coactivators and effectors of the Hippo signaling pathway (24), activate intestinal fibroblasts and exacerbate their fibrotic phenotype in chronic intestinal inflammation. High expression of YAP/TAZ can contribute to intestinal obstruction in CD patients (25). Additionally, several inflammatory cytokines, such as IL-1β, TNF-α, PDGF, and IL-36, can stimulate intestinal fibroblasts and aggravate the fibrotic phenotype (26, 27). Therefore, we first constructed an endoscopic prediction model for SES-CD ≥8, which could reflect the degree of intestinal inflammation to provide a more direct and effective approach to predict intestinal obstruction to some extent.

This study has several limitations. First, missing follow-ups and incomplete clinical data existed because of the retrospective nature of the study. Second, further studies with larger sample size and longer follow-up period should be performed to confirm the results.

## Conclusion

In this study, we identified the most significant threshold evaluation of SES-CD and demonstrated that an SES-CD score of 8 had the best predictive value for intestinal obstruction. We further indicated that SES-CD ≥8 was the most risk contributing factor for intestinal obstruction and significantly decreased the intestinal obstruction-free survival time. Thus, we constructed an endoscopic prediction model for SES-CD ≥8, which outperformed the clinical disease activity and could be an effective predictor of intestinal obstruction in CD. More frequent follow-up and colonoscopic surveillance should be considered in patients with SES-CD ≥8 to prevent the development of intestinal obstruction.

## Data Availability

The raw data supporting the conclusions of this article will be made available by the authors, without undue reservation.
