# Supplementary material for: The endoscopic prediction model of simple endoscopic score for Crohn’s disease (SES-CD) as an effective predictor of intestinal obstruction in Crohn’s disease: A multicenter long-term follow-up study
Source: Front Surg. 2022 Sep 9;9:984029. doi: 10.3389/fsurg.2022.984029 (PMC9632952; doi:10.3389/fsurg.2022.984029)
Supplement: Supplementary file 1 [file Datasheet1.docx]

| **Supplementary Table 1. Definitions of Simple Endoscopic Score for Crohn's Disease（SES-CD）** | | | |  |
| --- | --- | --- | --- | --- |
|  |  | **Simple Endoscopic Score for Crohn's Disease values** | |  |
| **Variable** | **0** | **1** | **2** | **3** |
| Size of ulcers | None | Aphthous ulcers (Ø <0.5 cm) | Large ulcers (Ø 0.5 to 2 cm) | Very large ulcers (Ø >2 cm) |
| Area of ulcers | None | <10% | 10-30% | >30% |
| Affected range | None | <50% | 50-75% | >75% |
| Intestinal stenosis | None | Single, endoscope can be passed | Multiple, endoscope can be passed | Endoscope cannot be passed |
| Ø, Diameter. |  |  |  |  |

| **Supplementary Table 2. Definitions of Crohn’s Disease Activity Index (CDAI)** | | | | | | |  |  |  |  |  |  |  |
| --- | --- | --- | --- | --- | --- | --- | --- | --- | --- | --- | --- | --- | --- |
|  | **Variable** |  |  |  |  | **Day** |  |  |  |  | **7 Day** | **Weighting** | **Total** |
|  |  |  | **1** | **2** | **3** | **4** | **5** |  | **6** | **7** | **Total** | **Factor** |  |
| **1. Number of liquid or very soft stools** | | |  |  |  |  |  |  |  |  |  | x 2= |  |
| **2. Abdominal pain** | |  |  |  |  |  |  |  |  |  |  | x 5= |  |
| 0=none, 1=mild, 2=moderate, 3=severe | | |  |  |  |  |  |  |  |  |  |  |  |
| **3. General well-being** | |  |  |  |  |  |  |  |  |  |  | x 7= |  |
| 0=generally well, 1=slightly under par, | | |  |  |  |  |  |  |  |  |  |  |  |
| 2=poor, 3=very poor, 4=terrible | | |  |  |  |  |  |  |  |  |  |  |  |
| **4. Extra-intestinal manifestations, Current** | | | |  |  |  |  |  |  |  | **Check the existing condition** | | |
|  |  |  | Ⅰ. Arthritis/arthralgia | |  |  |  |  |  |  |  |  |  |
|  |  |  | Ⅱ. Iritis/uveitis | |  |  |  |  |  |  |  |  |  |
|  |  |  | Ⅲ. Erythema nodosum,pyoderma gangrenosum,aphthous stomatitis | | | | |  |  |  |  |  |  |
|  |  |  | Ⅳ. Anal fissure,fistula,or abscess | | |  |  |  |  |  |  |  |  |
|  |  |  | Ⅴ. Other fistula | |  |  |  |  |  |  |  |  |  |
|  |  |  | Ⅵ. Fever over 37.8℃ during past 7 days | | | |  |  |  |  |  |  |  |
|  |  |  |  |  |  |  |  |  |  | Total number of checked boxes= | | |  |
|  |  |  |  |  |  |  |  |  |  |  |  | x 20= |  |
| **5. Diphenoxylate, Opiates for diarrhea in the last 7 days** | | | | | |  |  |  |  |  | No=0, Yes=1 | |  |
|  |  |  |  |  |  |  |  |  |  |  |  | x 30= |  |
| **6. Abdominal mass** | |  |  |  |  |  |  |  | None = 0, Questionable = 2, Definite = 5 | | | |  |
|  |  |  |  |  |  |  |  |  |  |  |  | x 10= |  |
| **7. Local Haematocrit (%, take integer)** | | | | |  |  |  |  |  |  | If Male, 47- _____ = | |  |
|  |  |  |  |  |  |  |  |  |  |  | If Female,42- _____= | |  |
|  |  |  |  |  |  |  |  |  |  |  | 0 if negative | |  |
|  |  |  |  |  |  |  |  |  |  |  |  | x 6= |  |
| **8. Body weight calculation** | | | |  |  |  |  |  |  |  |  |  |  |
| If Male, standard weight(kg) = height(cm)-100 | | | | |  |  |  | (standard weight - actual weight) / standard weight= | | | | |  |
| If Female, standard weight(kg) = height(cm)-102 | | | | |  |  |  |  |  |  |  | x 100= |  |
|  |  |  |  |  |  |  |  |  |  |  | **CDAI TOTAL=** | |  |
